# Incidence of Thrombotic Complications in COVID-19 Patients and the Impact of Antithrombotic Therapy on ICU Mortality

**DOI:** 10.7759/cureus.77602

**Published:** 2025-01-17

**Authors:** Mohamed F Hendi, Zeyad F Alrais, Mohamed I Shoaib, Khalid M Hassan, Sulaiman M Zaifa

**Affiliations:** 1 Critical Care Medicine, Rashid Hospital, Dubai Academic Health Corporation, Dubai, ARE; 2 Critical Care Medicine, Mohammed Bin Rashid University of Medicine and Health Sciences, Dubai, ARE

**Keywords:** antiplatelet therapy, antithrombotic therapy, coronavirus disease 2019 (covid-19), covid-19 outbreak, thromboembolic events, thrombotic complication

## Abstract

Aim

We aimed to determine the incidence of thrombotic complications and outcomes of critically ill COVID-19 patients admitted to the intensive care unit (ICU) and evaluate the association between combined antithrombotic therapy and mortality in ICU patients admitted for COVID-19 pneumonia.

Methods

We retrospectively collected data of adult critically ill patients with COVID-19 admitted to the ICU in a major hospital in Dubai during the COVID-19 pandemic. The primary outcome was in-hospital mortality. Secondary outcomes included the incidence of major complications, such as thrombotic complications during the ICU stay. The study population was classified into two groups based on the type of prophylactic anticoagulant and antiplatelet therapy received.

Results

The study included 257 ICU patients admitted with COVID-19 pneumonia. The mean duration of their ICU stay was 24.95 days, ranging from one day to 327 days. The primary outcome was in-hospital mortality. In our study, 151 patients (58.7%) suffered in-hospital mortality.

Secondary outcomes included the incidence of major complications during the ICU stay. A total of 202 patients (78.6%) presented with acute respiratory distress syndrome. Ninety-nine (38.5%) of the patients had progressed to acute kidney injury. Thirty-three patients (12.8%) had various thrombotic complications. Three of these (9%) had venous thrombosis, and 30 patients (91%) had arterial thrombosis. Ischemic stroke was the major thrombotic complication of COVID-19 (36.3% of overall thrombotic events, n = 12), followed by myocardial infarction (27.2%; n = 9) and pulmonary embolism (21.2%; n = 7).

Out of 257 COVID-19 ICU patients, 73 patients (28.4%) received both anticoagulants and antiplatelet therapy, and 183 patients (70.8%) received only anticoagulant therapy. We compared the mortality of COVID-19 ICU patients who received anticoagulants alone to those with added antiplatelets. The application of combined antiplatelet and anticoagulants as thromboprophylaxis for COVID-19 ICU patients was not associated with a significant reduction in mortality (P = 0.868). Peak serum levels of D-dimer significantly correlate with the length of ICU stay (rho = 0.137, P = 0.031). Peak D-dimer level during the ICU stay was statistically significantly higher in non-survivors (mean = 11.87) compared to survivors (mean = 8.59) (P < 0.001). D-dimer on ICU admission had a good prognostic value for ICU patients with COVID-19 infection (P = 0.018).

Conclusion

The incidence of thrombotic complications among COVID-19 pneumonia patients admitted to ICU is remarkably high, which reinforces the recommendation to apply thrombosis prophylaxis strictly to all ICU patients admitted with COVID-19. The application of combined antiplatelets with anticoagulants as thromboprophylaxis for COVID-19 ICU patients was not associated with a significant reduction in ICU mortality. D-dimer has a significant correlation with prognosis and length of ICU stay of COVID-19 patients.

## Introduction

In January 2020, we were facing an outbreak of coronavirus disease 2019 (COVID-19) caused by a novel coronavirus (SARS-CoV-2). The COVID-19 pandemic had a significant public health impact, and there was a huge increase in demand for the critically ill service owing to the massive growth in the number of patients with severe illness. It is estimated that 5% to 20% of infected patients required hospital admission, of whom 5% to 15% developed critical illness requiring intensive care support [1].

Although COVID-19 primarily causes respiratory illnesses, clinical evidence has shown that severe COVID-19 patients can develop complications like venous thromboembolism and arterial thrombosis as well [2]. Early in the COVID-19 pandemic, the rate of thrombosis in hospitalized COVID-19 patients was identified as relatively high, especially in patients admitted to the ICU who were at the highest thrombotic risk [3].

During the COVID-19 pandemic, we reported many cases of thrombotic complications precipitated by COVID-19 infection in the ICU stays. We had to pay attention to common complications associated with COVID-19 pneumonia to reduce the associated mortality from the disease. Precise information about the incidence of thrombotic complications in COVID-19 patients is critical for determining the intensity of thromboprophylaxis [2].

This study focuses on issues related to COVID-19 complications in critically ill adult patients with COVID-19 pneumonia who were admitted to the ICU. We aimed to determine the incidence of thrombotic complications and outcomes of COVID-19 patients admitted to the ICU and evaluate the association of common antithrombotic therapy with ICU mortality.

## Materials and methods

Study design and ethical consideration

We conducted this retrospective cohort observational study on 257 COVID-19 pneumonia patients admitted between March 2020 and July 2021. Ethical approval was obtained from the Institutional Review Board, Dubai Ethical Committee (code number: DSREC-06-2020-36). Oral and nasal swabs were collected for SARS-CoV-2 polymerase chain reaction (PCR) during the pandemic for any suspected COVID-19 pneumonia patients.

Inclusion criteria

We enrolled in our study critically ill adult patients who were diagnosed with COVID-19 pneumonia and admitted to the ICU of a major hospital in Dubai during the COVID-19 pandemic. The study was conducted on adult patients who were aged at least 18 years and who had a SARS-CoV-2 infection confirmed by qualitative PCR of a nasal or oral swab.

Exclusion criteria

Patients who had a history of hypercoagulable disorder (thrombophilia), e.g., protein C, protein S, and antithrombin III deficiency, factor V Leiden (activated protein C resistance), prothrombin gene mutation, anti-phospholipid antibody syndrome, and sickle cell disease were excluded from our study.

Also, patients with a known history of coagulopathy (bleeding disorder), e.g., inherited hemophilia A and B, Von Willebrand disease, inherited clotting factor deficiencies, congenital platelet disorders, idiopathic thrombocytopenic purpura, and hepatic failure were excluded from our study.

Study population and data collection

We classified the study population into two groups based on who received dual antithrombotic therapy (prophylactic anticoagulant and antiplatelet therapy) and who received anticoagulant alone during ICU admission.

We collected epidemiological and clinical data and outcomes retrospectively from the hospital’s electronic medical record system using a designed audit tool. We calculated the incidence of major complications facing COVID-19 patients during ICU admission. We collected results of some laboratory tests conducted on the day of ICU admission; these included D-dimer, ferritin, lactate dehydrogenase (LDH), and also results of the highest peak level of those laboratories during the whole ICU stay via retrospective collection of laboratory flow charts from our electronic hospital file system. We analyzed the collected data retrospectively using Microsoft Excel 2016 (Microsoft Corporation, Redmond, WA). Informed consent was waived because of the retrospective nature of the study.

Statistical methods

We coded and entered data using SPSS version 28 (IBM Corp., Armonk, NY). We used frequency (count) and relative frequency (%) to summarize the categorical data, whereas for quantitative data, we used mean, median, standard deviation, minimum, and maximum.

We used the Mann-Whitney and nonparametric Kruskal-Wallis tests to compare quantitative variables [4]. We compared categorical data using the chi-square (c^2^) test. When the expected frequency was less than five, we utilized the exact test instead [5].

We used the Spearman correlation coefficient [6] to determine correlations between quantitative variables. The Spearman correlation coefficient (rho) shows the strength and direction of the correlation. If rho is positive, then the correlation direction will be positive; otherwise, the correlation direction will be negative, if rho is negative.

The amount of rho determines the correlation’s strength, irrespective of its sign. A weak correlation is present if the rho value is less than 0.3. A moderate correlation is present if the rho value is between 0.3 and 0.49. A strong correlation is present if the rho value is more than 0.5.

The receiver operating characteristic (ROC) curve was calculated to assess the relation between many laboratory tests, such as D-dimer, ferritin, LDH, and mortality. To determine the best cutoff value for detecting the outcome of significant parameters, we created an ROC curve and analyzed it using the area under the curve (AUC).

P-values less than 0.05 were regarded as statistically significant. P-values less than 0.001 were regarded as highly statistically significant.

## Results

The study was conducted on 257 ICU patients admitted with COVID-19 pneumonia. Our patients had a median age of 53 (22-92) years; 202 patients were male (78.6%). The mean duration of ICU stay was 24.95 days, ranging from one day to 327 days of ICU stay. The primary outcome was in-hospital mortality. In our study, 151 patients (58.7%) had in-hospital mortality, as shown in Table 1.

**Table 1:** Demographic data and outcomes of the studied population.

Demographic data		Count	%
Sex	Male	202	78.6%
	Female	55	21.4%
ICU outcome	Died	151	58.7%
	Discharged	106	41.3%
	Range	Mean ± SD	Median
Age (year)	22-92	53.80 ± 13.9	53.00
Length of ICU stay (day)	1-327	24.95 ± 35.81	15.00

Secondary outcomes included the incidence of major complications during the ICU stay, as shown in Table 2. A total of 202 patients (78.6%) presented with acute respiratory distress syndrome (ARDS). Ninety-nine (38.5%) of the patients had progressive acute kidney injury (AKI) during their ICU stays. Thirty-three patients (12.8%) had various thrombotic complications. Three of these (9%) had venous thrombosis, and 30 patients (91%) had arterial thrombosis.

**Table 2:** Distribution of common COVID-19 complications in the study.

Common complications of the COVID-19 infection in the ICU		Count	%
Acute respiratory distress syndrome (ARDS)	Yes	202	78.6%
	No	55	21.4%
Acute kidney injury (AKI)	Yes	99	38.5%
	No	158	61.5%
Septic shock	Yes	43	16.7%
	No	214	83.3%
Thrombotic events	Yes	33	12.8%
	No	224	87.2%
Pneumothorax	Yes	19	7.4%
	No	238	92.6%
Bleeding	Yes	13	5%
	No	244	95%
Diabetic ketoacidosis (DKA)	Yes	9	3.5%
	No	248	96.5%

**Figure 1:**
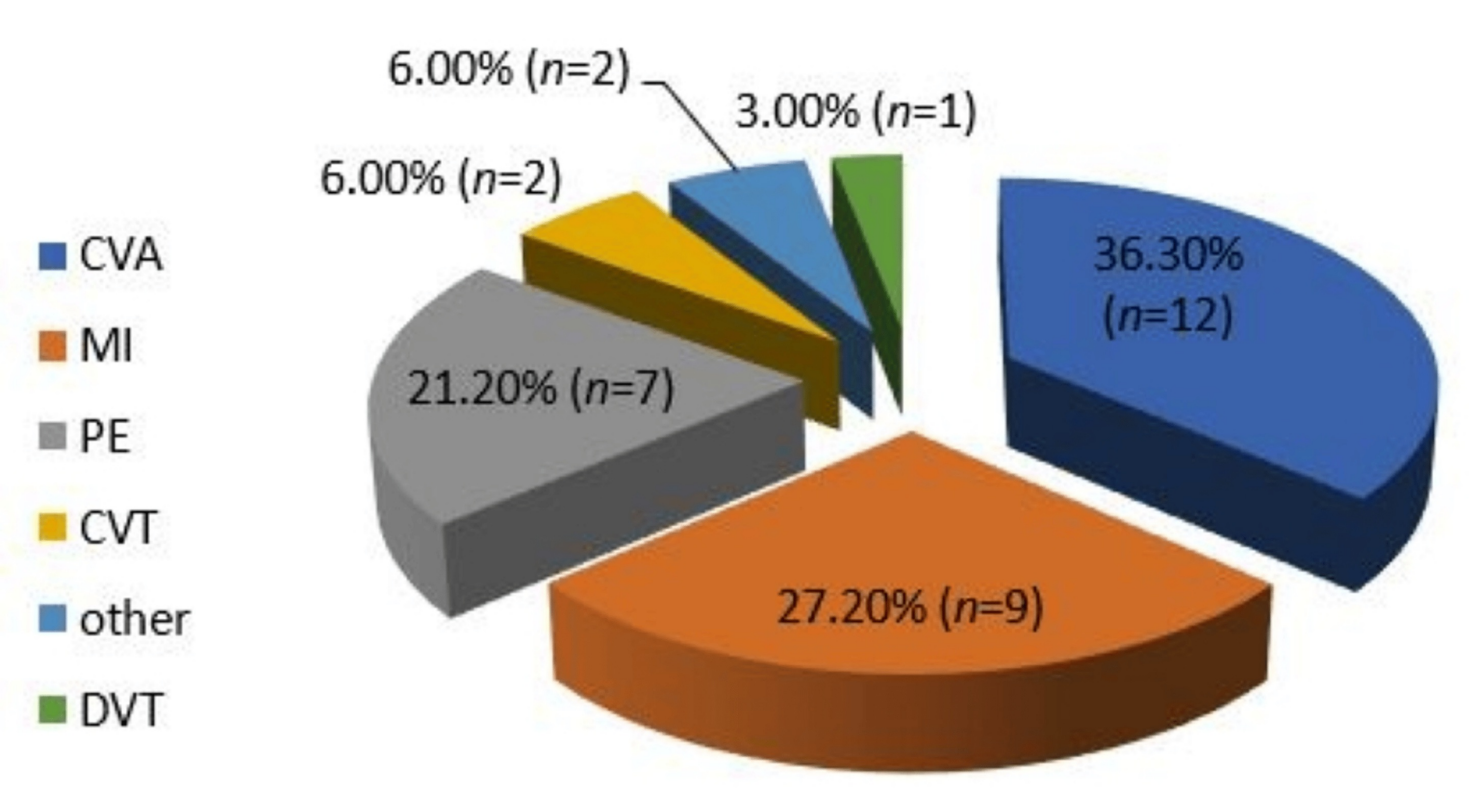
Distribution of thrombotic complications among the studied population. CVA: cerebrovascular accident; MI: myocardial infarction; PE: pulmonary embolism; CVT: cavernous sinus thrombosis; DVT: deep venous thrombosis.

The incidence of thrombosis was noted in 12.8% of the studied population (33 patients). Ischemic stroke (cerebrovascular accident) was the major thrombotic complication of COVID-19 (36.3%), followed by myocardial infarction (MI; 27.2%) and pulmonary embolism (PE; 21.2%). Other thrombotic events have been reported, e.g., bowel ischemia, peripheral artery thrombosis, cavernous sinus thrombosis (CVT), and deep venous thrombosis (DVT), as shown in Figure 1.

Out of 257 COVID-19 ICU patients during the study period (two patients were excluded because they received no antithrombotic therapy), 73 (28.4%) patients received both anticoagulants and antiplatelet therapy, and 183 (70.8%) patients received only anticoagulant therapy as thromboprophylaxis, as shown in Table 3.

**Table 3:** Mortality and discharge distribution among the studied groups and P-value analysis via the chi-square test.

		Group				
		Anticoagulant and antiplatelets		Anticoagulant		
		Count	%	Count	%	P-value
	Total	73	28.4%	182	70.8%	
Outcome	Death	45	61.6%	106	58.2%	0.868
	Discharge	28	38.4%	76	41.8%	

We compared the mortality of COVID-19 ICU patients who received standard prophylactic anticoagulants alone to those who also received an antiplatelet agent, e.g., aspirin. The application of an antiplatelet agent combined with anticoagulants for COVID-19 ICU patients was not associated with a significant reduction in ICU mortality (P-value = 0.868), as shown in Table 3.

We noted that the peak D-dimer and ferritin levels during ICU stays were statistically significantly higher in non-survivors (with means of 11.87 and 7836, respectively) compared to survivors (means of 8.59 and 2095, respectively), with a P-value < 0.001, as shown in Table 4.

**Table 4:** Mean D-dimer levels (peak and on admission) among survivors and non-survivors and P-value analysis via Mann-Whitney tests. LDH: lactate dehydrogenase; O/A: on admission.

	Outcome										
	Death (non-survivors)					Discharge (survivors)					P-value
	Mean	SD	Median	Minimum	Maximum	Mean	SD	Median	Minimum	Maximum	
Ferritin O/A	2087.79	4130.03	1288.00	18.00	43678.00	1280.76	2286.46	954.85	20.80	21890.00	0.001
Ferritin peak	7838.26	19518.34	2626.50	72.00	151800.00	2095.68	2466.11	1681.50	94.00	21890.00	<0.001
D-dimer O/A	5.02	6.30	1.98	0.40	20.00	3.37	5.05	1.50	0.20	20.00	0.031
D-dimer peak	11.87	7.11	12.53	0.68	20.00	8.59	7.18	4.95	0.98	20.00	<0.001
LDH O/A	511.25	225.45	490.00	141.00	1572.00	501.59	579.22	406.50	94.00	5732.00	0.013
LDH peak	898.25	1454.87	648.00	141.00	12512.00	691.00	577.72	632.00	241.00	5732.00	0.165

Also, there was a statistical difference in serum D-dimer, ferritin, and LDH levels upon admission (O/A) between non-survivors (means of 5.02, 2087, and 511.25, respectively) and survivors (means of 3.37, 1280, and 501.6, respectively) with P-values of 0.031, 0.001, and 0.013, respectively.

**Figure 2:**
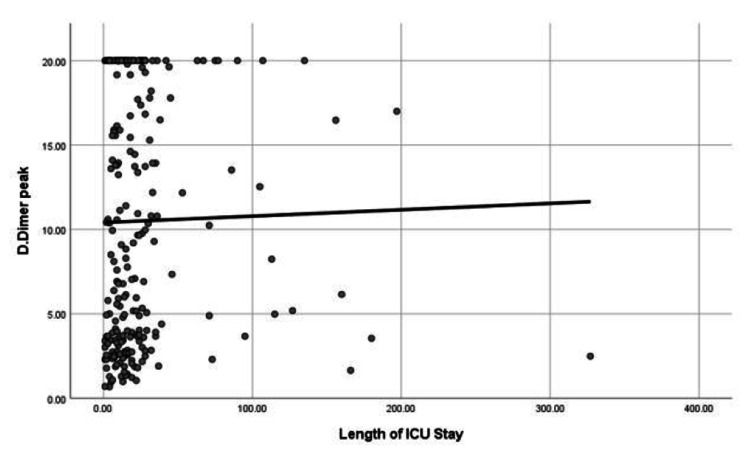
Correlation between peak D-dimer level and length of ICU stay.

Our data showed that peak serum levels of D-dimer positively correlated to the length of ICU stay (correlation coefficient; rho = 0.137, P-value = 0.031), as shown in Figure 2.

**Figure 3:**
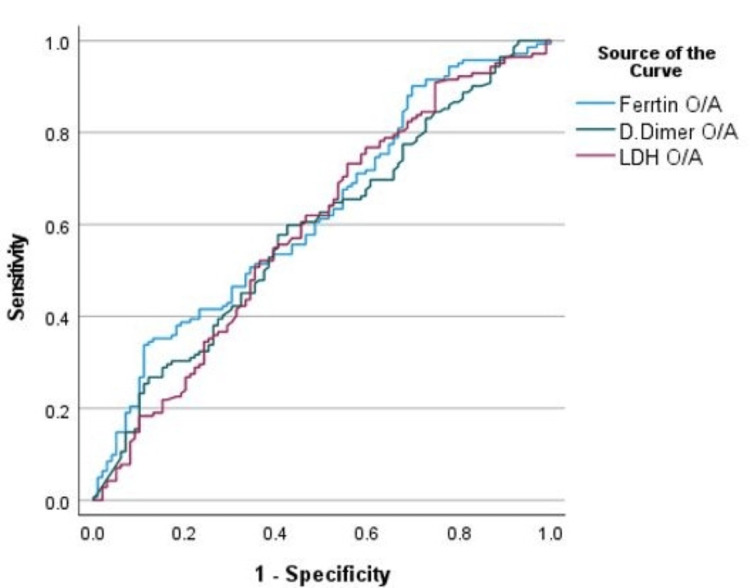
ROC curve for serum D-dimer, ferritin, and LDH levels on admission as prognostic markers of mortality in COVID-19 infection in the ICU. ROC: receiver operating characteristic; LDH: lactate dehydrogenase; O/A: on admission.

The ROC curve was calculated to assess the relation between various laboratory tests (e.g., D-dimer, ferritin, and LDH) and mortality, as shown in Figure 3. We noted that some laboratory results on admission (e.g., D-dimer, ferritin, and LDH) were significant predictors of mortality of COVID-19 patients, as shown in Table 5.

**Table 5:** Prognostic ability of serum D-dimer, ferritin, and LDH (peak and on admission levels) in COVID-19 infection via ROC curve analysis. ROC: receiver operating characteristic; LDH: lactate dehydrogenase; O/A: on admission; PPV: positive predictive value; NPV: negative predictive value.

Mortality	Area under the curve	P-value	95% confidence interval		Cutoff value	Sensitivity %	Specificity %	PPV %	NPV %	Accuracy %
			Lower bound	Upper bound						
Ferritin O/A	0.624	0.001	0.552	0.695	1297.5	50.7	65.7	68.47	47.89	56.92
D-dimer O/A	0.588	0.018	0.515	0.661	1.61	59.9	57.6	67.16	49.58	58.89
LDH O/A	0.592	0.015	0.518	0.666	456.5	54.9	60.6	66.67	47.69	56.92
Ferritin peak	0.691	<0.001	0.626	0.757	2598.5	52.1	79	78.00	52.94	62.85
D-dimer peak	0.635	<0.001	0.564	0.707	7.935	64.8	62	71.32	54.70	63.64
LDH peak	0.556	0.131	0.483	0.629	----	-----	-----	----	----	-----

## Discussion

During the COVID-19 pandemic, there was a huge increase in demand for critically ill service owing to the massive growth in the number of patients with severe illness. We should pay more attention to common complications of COVID-19 to reduce the associated mortality from the disease.

Although COVID-19 primarily causes respiratory illnesses, clinical evidence has shown that severe COVID-19 patients can develop complications like venous thromboembolism and arterial thrombosis as well. Diffuse intravascular coagulation, hypoxia, immobility, and excessive inflammation are some of the reasons that COVID-19 may increase the risk of both arterial and venous thromboembolic disease [2].

Early in the COVID-19 pandemic, the rate of thrombosis in hospitalized COVID-19 patients was identified as relatively high, especially in patients admitted to the ICU who were at the highest thrombotic risk [3]. We reported many cases of thrombotic complications precipitated by COVID-19 infection during ICU stays. Precise information about the incidence of thrombotic complications in COVID-19 patients is critical for determining the intensity of thromboprophylaxis [2].

One of the grave consequences of COVID-19 infection is a prothrombotic condition state that manifests as micro-thrombotic, thrombotic, and thromboembolic events. In severe infections, COVID-19 itself causes a hypercoagulable state by changing the natural balance of circulating prothrombotic factors [7].

Hypercoagulability can be thought of in terms of Virchow’s triad (venous stasis, vascular injury, and hypercoagulability), given that all three of the major contributions to clot formation apply to severe COVID-19 infection. Stasis occurs when critically ill patients are immobilized, causing blood flow stasis. Also, endothelial injury occurs when the SARS-CoV-2 virus directly invades endothelial cells, potentially causing cell injury. Endothelial injury can also be caused by intravascular catheters and acute systemic inflammatory response mediators, including cytokines (e.g., interleukin 6) and other acute phase reactants.

In patients with severe COVID-19, several alterations in circulating prothrombotic factors have been documented that lead to hypercoagulability. Very elevated levels of D-dimer have been observed that correlate with illness severity; D-dimer is a degradation product of cross-linked fibrin indicating augmented thrombin generation and fibrin dissolution by plasmin. However, high D-dimer levels are common in acutely ill individuals with a number of infectious and inflammatory diseases [8]. Therefore, it is important to comprehend how antiplatelet and anticoagulant therapy affect COVID-19 treatment [7].

In our study, we aimed to determine the incidence of thrombotic complications and outcomes of COVID-19 patients admitted to the ICU to highlight the role of thrombosis in COVID-19 and evaluate the use of common antithrombotic therapy with ICU mortality. Accurately quantifying thrombotic risk may assist with prognosis and guide appropriate thromboprophylaxis.

We carried out this study on 257 critically ill patients admitted to the ICU with COVID-19 pneumonia. Age ranged from 22 to 92 years old (mean = 53.8). The mean duration of ICU stay was 24.95 days, ranging from one day to 327 days of ICU stay. We noticed that males represented 78.6% (n = 202) of our study population, which can be explained by the fact that 90% of the Dubai population consists of expats.

Regarding the incidence of major complications during ICU stays in our study, 202 patients (78.6%) presented with ARDS. Ninety-nine (38.5%) of the patients had progressed to AKI. Thirty-three patients (12.8%) had various thrombotic complications. Three of these (9%) had venous thrombosis and 30 patients (91%) had arterial thrombosis.

The incidence of venous thromboembolic events (VTEs) in COVID-19 patients varies depending on the patient population (e.g., ICU or non-ICU patients). Nopp et al., in a meta-analysis of 66 observational studies, reported an overall VTE prevalence of 14.1% in 28,173 hospitalized patients with COVID-19 (mean age of 62.6 years, 60.1% men, 19.4% ICU patients). The prevalence was higher in ICU patients at 22.7% (45.6% with a screening ultrasound (US) and 18.7% without screening) compared with 7.9% in non-ICU patients [9,10].

In our study, the incidence of thrombosis was 12.8% of the studied population (33 patients). Ischemic stroke was the major thrombotic complication of COVID-19 (36.3%, n = 12), followed by myocardial infarction (27.2%, n = 9) and PE (21.2%, n = 7). Other thrombotic events had been reported in our study (e.g., bowel ischemia, peripheral artery thrombosis, cavernous sinus thrombosis, and deep venous thrombosis).

Lodigiani et al. reported on 388 patients with COVID-19 (the mean age was 66 years; 68% were men, and 16% required ICU), and found that VTEs occurred in 28 patients (7.7% of closed cases). Of these, 27.6% were in the ICU, 6.6% were in the general ward, and PE was confirmed in 10 patients. The rate of myocardial infarction and ischemic stroke was 1.1% and 2.5%, respectively. They included critical and critically ill patients. Thromboprophylaxis was used for 100% of ICU patients and 75% of those in the general ward. They reported a high number of venous and arterial thromboembolic events diagnosed within 24 hours of admission, with a high rate of positive VTE imaging tests among the few COVID-19 patients tested [11].

There is a high incidence of thrombotic complications in critically ill COVID-19 patients. VTE is the most common thrombotic complication and has been observed in up to 23% of critically ill COVID-19 patients. However, critically ill patients may be at increased risk for bleeding complications, which may also occur in patients with critical illness related to COVID-19. The optimal strategy is thromboprophylaxis, which balances the risks of bleeding and thrombosis [10].

Thromboprophylaxis is widely used for most ICU patients because it has a net therapeutic benefit when balancing efficacy to prevent thrombosis and safety (risk of bleeding), whereas mechanical methods of thromboprophylaxis are recommended only in a minority of patients with a high bleeding risk [12]. Risk factors for bleeding are patient-specific factors that include age, underlying disease severity (e.g., sepsis-associated coagulopathy), and comorbidities (e.g., impaired hepatic or renal function) [13].

Coagulation abnormalities in COVID-19 patients are clearly correlated with poor prognosis. In our study, 151 patients (58.7%) had in-hospital mortality. The thromboembolic risk in COVID-19 patients has led some experts to suggest a more aggressive thromboprophylaxis dosing of anticoagulants or even empiric therapeutic dose anticoagulation for VTE prevention.

Aggressive thromboprophylaxis versus standard prophylactic thromboprophylaxis

Thromboprophylaxis has been used as one of the most essential therapeutic approaches for COVID-19 patients since the onset of the COVID-19 pandemic [13]. Low molecular weight heparin (LMWH, e.g., enoxaparin) represents the most well-studied type of thromboprophylaxis in hospitalized patients [13].

A multiplatform trial (MPT) incorporated three multicenter studies (REMAP-CAP, ATTACC, and ACTIV-4A) of critically ill patients with COVID-19 infection [14], and compared prophylactic with therapeutic intensity anticoagulation [14] to address the question of whether therapeutic anticoagulation is indicated in critically ill COVID-19 patients [14].

The MPT trial initially evaluated the therapeutic dose versus institutional standard prophylaxis regimens (including both intermediate and standard prophylactic doses of heparin) in 1,098 critically ill patients with severe COVID-19 infection who were randomly assigned to receive either therapeutic anticoagulation with heparin (529 patients) or standard prophylactic regimens as per local usual care (545 patients). Hospital survival was nonsignificant between the therapeutic anticoagulant group and the standard care pharmacological thromboprophylaxis group (64.3% vs. 65.3%, respectively). The MPT trial concluded that therapeutic anticoagulation did not improve hospital survival or increase the number of days free of organ support compared to standard pharmacological thromboprophylaxis in patients with severe COVID-19 [14].

A second randomized trial, the ACTION trial by Lopes et al., compared therapeutic intensity versus prophylactic intensity anticoagulation in hospitalized patients with COVID-19 and elevated D-dimer. Most of the patients in the ACTION trial were not critically ill. Therapeutic intensity anticoagulation with enoxaparin or heparin (for the unstable patients) or rivaroxaban (for the stable patients) did not improve clinical outcomes and was associated with increased bleeding [15]. Lopes et al. concluded that for patients hospitalized with COVID-19 infection and elevated D-dimer levels, therapeutic anticoagulation during the hospital stay did not improve clinical outcomes and increased bleeding risk compared with prophylactic anticoagulation. Therefore, the use of therapeutic dose rivaroxaban and other direct oral anticoagulants should be avoided for individuals in the absence of any evidence-based indication for oral anticoagulation [15].

Based on these previous trials, we included advice in our hospital policy that in critically ill patients hospitalized for COVID‐19, a therapeutic dose of anticoagulants (LMWH/unfractionated heparin (UFH)) is not recommended over the standard prophylactic dose of LMWH/UFH, because increased doses of LMWH/UFH do not provide any benefit for preventing progression of COVID-19 or death. Also, the American Society of Hematology guideline panel currently suggests using prophylactic intensity over intermediate intensity or therapeutic intensity anticoagulation in critically ill COVID-19 patients, especially ICU patients who do not have confirmed or suspected VTE [10].

Antithrombotic therapy as thromboprophylaxis in COVID-19

LMWH is the preferred anticoagulant for hospitalized COVID-19 patients, according to a recent guidance statement issued by the International Society on Thrombosis and Haemostasis (ISTH) [16].

According to the guidance statement, add-on treatment with antiplatelet therapy is potentially harmful and not advised for thromboprophylaxis in the general population of COVID-19 [17,18]. The exception could be in critically ill COVID-19 patients who have a low bleeding risk and who have been treated with prophylactic doses of LMWH and proton pump inhibitors for gastric protection [13].

Our study aimed to identify whether concurrent use of antiplatelet and anticoagulants was associated with an improved outcome in critically ill COVID-19 patients. We classified the study population into two groups based on who received dual antithrombotic therapy (prophylactic anticoagulant and antiplatelet therapy) and who received anticoagulant alone during ICU admission to evaluate the association of common antithrombotic therapy with ICU mortality.

Previous clinical studies evaluated the role of antiplatelet drugs in various conditions of COVID-19 patients [17-19]. Our study is different because it was only concerned with the use of a standard dose of LMWH with aspirin as an antiplatelet treatment for critically ill ICU patients with COVID-19. It should be highlighted that in these previous studies, hospitalized patients were already receiving anticoagulation for thromboprophylaxis in varying doses.

Antithrombotic for nonhospitalized outpatients

A randomized trial of 657 nonhospitalized symptomatic outpatients with COVID-19 conducted by Connors et al. showed no benefit of initiation of antiplatelet therapy (aspirin 81 mg) or apixaban after diagnosis of COVID‐19 in the outpatients, and there was no significant difference between the placebo group and the active groups in reduced risk of hospitalization, venous or arterial thrombosis, or mortality [20].

This study did not support the use of apixaban or aspirin for outpatients to reduce the major adverse pulmonary or cardiovascular consequences associated with symptomatic but clinically stable COVID-19 infection [21].

Yuan et al. studied 183 adult patients with coronary artery disease diagnosed with COVID‐19, including 52 taking low‐dose aspirin and 131 without using aspirin. This study reported that there was no difference in in-hospital mortality between the non‐aspirin group and the aspirin group (21.1% vs. 22.2%, P-value = 0.885). This study recommended that the pre‐hospitalization use of low-dose aspirin was not associated with improved outcomes of coronary artery disease patients hospitalized with COVID‐19 [22].

Antithrombotic for hospitalized noncritically ill patients

Two previous studies revealed no mortality benefit of antiplatelet therapy (including aspirin and P2Y12 inhibitors) as an add-on therapy among hospitalized noncritically ill COVID‐19 patients [17,18].

Berger et al. conducted a randomized clinical study with 562 noncritically ill patients admitted for COVID-19 to assess the benefits and risks of adding a P2Y12 inhibitor (clopidogrel was used in 37% and ticagrelor in 63%) to anticoagulant therapy among hospitalized noncritically ill COVID-19 patients. They concluded that when compared to a therapeutic dose of heparin alone, using a P2Y12 inhibitor in addition to heparin did not improve the number of organ support-free days throughout the 21-day hospital stay for noncritically ill COVID‐19 patients [17].

Another very large trial, RECOVERY, randomized 14,892 COVID-19 adult patients. Patients were randomly allocated (1:1) to receive aspirin (150 mg daily) or only standard care (LMWH/UFH). Patients allocated to aspirin had a slightly higher rate of discharge from the hospital alive within 28 days (75% vs. 74%; P-value = 0.0062). Among patients receiving invasive or noninvasive ventilation (N = 4920), there was no reduction in mortality risk within 28 days for the aspirin group compared to the control group (P-value = 0.35) [18].

The RECOVERY trial found that aspirin was associated with a slight increase in the likelihood of being discharged alive within 28 days of hospital stay, but not with a decrease in 28-day mortality or the risk of progression to invasive mechanical ventilation in noncritical, hospitalized COVID-19 patients [18].

However, these two studies also indicated evidence of harm with an increased risk of major bleeding events in patients on antiplatelet therapy [17,18].

Wang et al. (2024) conducted a study of 4481 hospitalized patients with confirmed COVID-19 and assessed the in-hospital complications risk of oral anticoagulants (OAC) or antiplatelets, respectively. They reported that 690 (14.1%) patients received OAC with or without antiplatelets (the most frequently used OAC was rivaroxaban), which was associated with reduced in-hospital mortality (P = 0.017) and reduced need for mechanical ventilation (P-value = 0.028) in COVID-19 patients compared with those who did not receive any antithrombotic medication; 4,191 patients (85.9%) received no antithrombotic therapy [23].

Antithrombotic hospitalized critically ill patients

A small study by Viecca et al. [24] enrolled five patients with severe respiratory failure with laboratory-confirmed SARS-CoV-2 infection, and another five patients formed the control group. They found that aspirin did not reduce mortality compared with the non‐aspirin group; however, they suggested that antiplatelet treatment may improve the ventilation/perfusion ratio in COVID-19 patients with severe respiratory failure [24], though the sample size of this study was found to be very small compared with our study.

In a large trial (REMAP‐CAP), 1557 critically ill adult patients with COVID-19 were enrolled from 105 sites in eight countries. Patients were randomized to either receive a P2Y12 inhibitor (n = 455; mainly clopidogrel at 75 mg daily), aspirin 75-100 mg daily (n = 565), or no antiplatelet agent (control group, n = 529), in addition to standard anticoagulation thromboprophylaxis. Most patients (90%) also received LMWH, whereas for 72%, the dose of thromboprophylaxis was low (prophylactic) or intermediate [19].

Results from the two antiplatelet groups were pooled and compared to the control group. In the REMAP‐CAP trial, the proportion of patients who survived to hospital discharge was 71.5% (723/1011) in the antiplatelet groups and 67.9% (354/521) in the control group; the adjusted absolute difference was 5%. Major bleeding occurred in 2.1% and 0.4% of patients in the antiplatelet and control groups; the adjusted absolute risk increase was 0.8% (a 99.4% probability of harm) [19].

This trial concluded that among the critically ill hospitalized COVID-19 patients, treatment with an antiplatelet agent, compared to no antiplatelet agent, had a low likelihood of improving the number of organ support-free days within 21 days. However, even this small advantage was accompanied by an increased risk of major bleeding [19,25].

Our study differed from the REMAP‐CAP trial in that the results from the two antiplatelet groups (aspirin and clopidogrel) in the REMAP-CAP trial were pooled and compared to a control group, whereas in our study, aspirin (75-100 mg) was the antiplatelet agent added on to the standard prophylactic anticoagulant as thromboprophylaxis and compared to the non-antiplatelet group.

In our study, out of 257 COVID-19 ICU patients, 73 (28.4%) patients received both anticoagulants and antiplatelet therapy, and 183 (70.8%) patients received only an anticoagulant without an antiplatelet agent for VTE thromboprophylaxis.

We compared the mortality of COVID-19 ICU patients who received anticoagulants alone and those who received an additional antiplatelet agent (aspirin 75-100 mg daily). The application of combined antiplatelets with anticoagulants for COVID-19 ICU patients was not associated with a significant reduction in in-hospital mortality (P‑value = 0.868).

We conclude that in critically ill patients hospitalized for COVID‐19, adding an antiplatelet agent like aspirin to the prophylactic dose of UFH/LMWH is not well established. The minor nonsignificant increase in mortality (non-survivors accounted for 61.1% (n = 45/73) of the aspirin group compared to 58.6% (n = 106/182) of the non-aspirin group; P-value = 0.868) maybe due to increased bleeding risk.

The value of D-dimer for COVID-19 patients

When imaging is not immediately available for COVID-19 patients to confirm or deny a diagnosis of PE or DVT, ICU physicians must depend on clinical assessments based on physical findings, history, and other tests. The possibility of PE is moderate to high in patients with symptoms or signs of DVT, unexplained worsening respiratory status, unexplained tachycardia or hypotension, or risk factors for thrombosis (e.g., a history of thrombosis, hormonal therapy, or cancer) [10].

The value of the D-dimer test is the ability to rule out DVT and PE effectively because it produces low false negative rates if the D-dimer level is normal. However, it is crucial to understand that COVID-19 patients, particularly those in critical or severe conditions, have higher D-dimer levels, independent of the absence or presence of VTE or PE, so D-dimer generally cannot be used to diagnose VTE or PE [15].

It has been demonstrated that elevated D-dimer levels are linked to more severe COVID-19 infections. In some trials of anticoagulants, high D-dimer levels have been utilized to risk stratify the patients. Whether an elevated D-dimer level alone predicts a response is unclear, with trials showing variable results [10].

Our study found no positive correlation between the length of ICU stay and the D-dimer level (P-value = 0.556) on admission, which suggests that the length of the ICU stay will not be affected by COVID-19 severity on admission. However, the peak blood D-dimer level significantly correlates with the length of ICU stay (P‑value = 0.031).

We noted in our study that there was a statistical difference in serum D-dimer levels on admission between non-survivors (mean = 5.02) and survivors (mean = 3.37), with a P-value = 0.031. Also, the D-dimer peak level during the ICU stay was statistically significantly higher in non-survivors (mean = 11.87) compared to survivors (mean = 8.59), with a P-value < 0.001.

For more optimum results, we calculated the ROC curve to evaluate the relation between various laboratory test levels on ICU admission and mortality. We observed that some laboratory variables on admission (e.g., D-dimer) had a significant P‑value (P-value = 0.018) for predicting ICU mortality in COVID-19 patients. Also, the peak D-dimer level during ICU admission had a good prognostic value for ICU patients with COVID-19 infection (P-value < 0.001).

Limitations

Our retrospective study was conducted during the COVID-19 pandemic. Moreover, the change in COVID-19 dominant variants, the wide immunization status (natural immunity and increasing rates of vaccination), and the availability of antiviral therapies for outpatients may have reduced the thromboembolic risk for COVID-19 patients; thus, more studies are needed for the optimal management of thrombotic risk for vaccinated patients.

The study was limited because of the small number of COVID-19 patients admitted to our ICU during the pandemic. Our study, like almost all previous studies, is retrospective in nature. Future multicenter randomized clinical trials are needed to evaluate the efficacy of aspirin on critically ill COVID-19 patients to better define recommendations for clinical practice to lower COVID-19-related mortality from thrombotic complications. Indeed, more research is needed to understand the mechanism of COVID-19-induced thrombosis.

Almost all previous studies are retrospective in nature, designed to better define recommendations of clinical practice. Multicenter placebo-controlled randomized clinical trials with outcomes are needed to evaluate the efficacy of aspirin.

## Conclusions

The incidence of thrombotic complications among COVID-19 pneumonia patients admitted to the ICU is remarkably high, which reinforces the recommendation to apply thrombosis prophylaxis strictly to all ICU patients admitted with COVID-19. The combined application of an antiplatelet agent (aspirin) with standard prophylactic anticoagulants for COVID-19 ICU patients was not associated with a significant reduction in in-hospital mortality.

Antiplatelet therapy should not be routinely started for thromboprophylaxis of COVID-19 patients. Concurrent use with anticoagulants should be evaluated individually, taking into consideration the indication for the antiplatelet agent as well as each patient's thrombotic or bleeding risk. Therefore, higher levels of D-dimer during ICU stays in non-survivors of COVID-19 patients and D-dimer’s significant correlation with prognosis and length of ICU stay should be taken into consideration during thromboprophylaxis management of COVID-19 in ICUs. Thus, the D-dimer level needs close attention in ICU patients with COVID-19.
